# In Vitro and In Vivo Anti-Inflammatory Effects of Cannabidiol Isolated from Novel Hemp (*Cannabis sativa* L.) Cultivar Pink Pepper

**DOI:** 10.3390/molecules28186439

**Published:** 2023-09-05

**Authors:** Jong-Hui Kim, Min Hong, Joon-Hee Han, Byeong Ryeol Ryu, Young Seok Lim, Jung Dae Lim, Chang Hyeug Kim, Soo-Ung Lee, Tae-Hyung Kwon

**Affiliations:** 1 Institute of Biological Resources, Chuncheon Bioindustry Foundation, Chuncheon 24232, Republic of Korea; jonghe5820@cbf.or.kr (J.-H.K.); fabre_min@cbf.or.kr (M.H.); cbfhjh@cbf.or.kr (J.-H.H.); kchyeug@cbf.or.kr (C.H.K.); 2 Department of Bio-Health Convergence, Graduate School, Kangwon National University, Chuncheon 24341, Republic of Korea; fbqudfuf0419@kangwon.ac.kr (B.R.R.); potatoschool@kangwon.ac.kr (Y.S.L.); ijdae@kangwon.ac.kr (J.D.L.); 3 Department of Bio-Health Technology, Kangwon National University, Chuncheon 24341, Republic of Korea; 4 Department of Herbal Medicine Resource, Kangwon National University, Samcheok 25949, Republic of Korea

**Keywords:** anti-inflammatory activity, cannabidiol, *Cannabis sativa*, edema, RAW 264.7 cells

## Abstract

*Cannabis sativa* L. contains more than 80 cannabinoids, among which cannabidiol (CBD) is the main neuroactive component. We aimed to investigate the anti-inflammatory efficacy of CBD in vitro and in vivo isolated from “Pink pepper”, a novel hemp cultivar, by repeating the method of selecting and cultivating individuals with the highest CBD content. We investigated the effects of CBD on inflammatory markers elevated by lipopolysaccharide (LPS) treatment in RAW 264.7 mouse macrophage cells through Western blot and RT-PCR. In addition, we confirmed these effects through the ELISA of inflamed paw tissue of a λ-carrageenan-induced mouse edema model that received an oral administration of CBD. CBD inhibited the LPS-induced phosphorylation of NF-κB and MAPK in RAW 264.7 and exhibited anti-inflammatory effects by participating in these pathways. In our in vivo study, we confirmed that CBD also inhibited the inflammatory mediators of proteins extracted from edematous mouse paw tissue. These results show that CBD isolated from “Pink pepper” exhibits potent anti-inflammatory effects. These anti-inflammatory effects of CBD have pharmacological and physiological significance, highlighting the industrial value of this novel cultivar.

## 1. Introduction

Inflammation is a biological response of the immune system that can be induced by various factors, including pathogens and damaged cells, and is an essential response to stimuli such as toxins, pathogens, and tissue damage [1,2]. However, excessive inflammation can become a major cause of unresolved diseases such as cancer, atherosclerosis, asthma, and multiple sclerosis [2,3]. Lipopolysaccharide (LPS) is a major component constituting the outer membrane of Gram-negative bacteria and induces an inflammatory response by stimulating the Toll-like receptor 4 (TLR4) in the macrophage cell membrane [4,5]. Macrophages stimulated with LPS are known to activate nuclear factor-kappa B (NF-κB) and mitogen-activated protein kinases (MAPK) signaling pathways through the activation of TLR4 [2]. Activated MAPKs and NF-κB express inflammatory response mediators, such as inducible nitric oxide synthase (iNOS) and cyclooxygenase-2 (COX-2), and induce the production of cytokines, such as interleukin-1β (IL-1β) and interleukin-6 (IL-6), and tumor necrosis factor α (TNF-α) [2,6].

*Cannabis sativa* L. has been cultivated globally for centuries for various purposes [7], and the pharmacological use of *Cannabis sativa* L. has been recorded in the oldest Chinese pharmacopoeia, *Shen Nung Pen Ts’ao Ching* [7,8]. Iftikhar et al. stressed the importance of *Cannabis sativa* L. in traditional medicine and reported that it was traditionally used for 210 ailments, including digestive and psychological disorders, pain, inflammation, and injuries [9]. In particular, *Cannabis sativa* L. was used as an ancient anti-inflammatory drug to reduce neuro-, wound-, and periodontal inflammation, emphasizing its pharmacological necessity [9,10,11].

Cannabis contains more than 80 cannabinoids, including tetrahydrocannabinol (THC), a psychotropic component [12], and cannabidiol (CBD), the main neuroactive component [13]. Owing to the addictive nature of THC, *Cannabis* has been restricted from widespread use for a long time. However, interest has been increasing in industrial hemp (*Cannabis sativa* L.), which contains higher levels of CBD compared to THC, and can be utilized as building material, grain, fiber, and oil without causing psychological dependence [12,13]. Hemp with a high CBD content does not contain THC and is evaluated as a sustainable and ecologically positive crop, with the possibility of utilizing the beneficial aspects of hemp without psychological dependence symptoms [13]. 

In this study, we experimented using a “Pink pepper”, a new Korean hemp variety with a high CBD content. This particular cannabis cultivar is bred and cultivated in Korea for medical use, with high levels of CBDA, low levels of Δ9-THCA, and auto-flowering (capable of flowering without the need for light cycle regulation), and can grow well both indoors and outdoors. It reaches heights of 207.5 ± 10.3 cm in open fields, 97.1 ± 10.1 cm in house pots, and 58.1 ± 9.9 cm under indoor hydroponic conditions. The assembled genome of the pink pepper variety has been deposited in GenBank under the accession number GCA_029168945.1 [14]. Annotated information, gene structure, and functional predictions of the genome are available in the Figs Share database (https://doi.org/10.6084/m9.figshare.21391449, accessed on 25 October 2022) [15].

**Figure 1:**
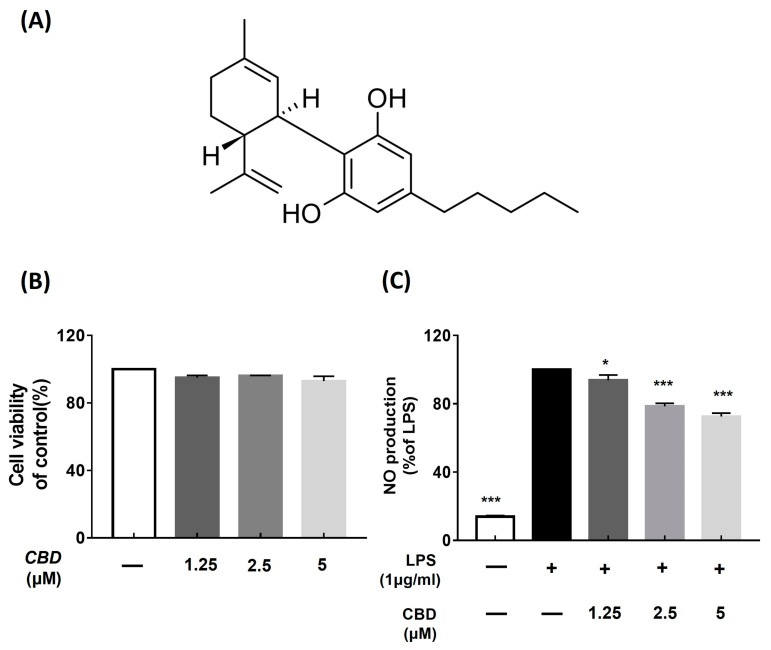
Structure of cannabidiol (CBD) and effect of CBD on LPS-induced nitric oxide (NO) generation and cell viability in RAW 264.7 cells. (**A**) Chemical structure of CBD; (**B**) viability of RAW 264.7 cells treated with various concentrations of CBD compared with that of the control; (**C**) results of inhibition of NO production rate in groups treated with various concentrations of CBD compared with that in LPS-only treatment group. Data represent the means ± SD in triplicate. *p*-value was calculated based on LPS-only data using ANOVA and Tukey’s post hoc test (* *p* < 0.05, *** *p* < 0.001).

Cannabidiol (CBD) is the decarboxylated form of the basic structure of cannabinoids, which are terpenophenolics comprising a diphenol and a monoterpene moiety (Figure 1A) [16]. CBD studies have examined the treatment of chronic pain [17], acute pain [18], and spasticity due to multiple sclerosis or paraplegia [19,20], as well as sleep issues and insomnia [12,21]. In addition, extensive studies have been conducted on the efficacy of CBD in anxiety disorders [22,23], and its anticancer effects have been reported in both in vitro and in vivo studies [16,24,25]. CBD showed effects on the cardiovascular system [26]. In addition, the evidence shows potential therapy for diabetes [27], antimicrobial potential [28], and remedies for COVID-19 [29]. The immunosuppressive and anti-inflammatory effects of CBD have been extensively studied for a long time, and the effects of CBD on the inhibition of cytokines through receptors stimulated by CBD have been reported [30,31]. However, not all the biological effects of CBD are fully understood, highlighting the need for ongoing research. 

Therefore, we confirmed the anti-inflammatory efficacy of CBD isolated from Korean new hemp “Pink Pepper” in vitro, using RAW 264.7 cells for the inflammatory response induced by LPS and evaluated the protein and gene mechanism. In addition, whether CBD shows efficacy in acute inflammation was investigated in vivo using a carrageenan-induced paw edema mouse model. This study aimed to investigate the effect of CBD (cannabidiol) isolated from new hemp “Pink Pepper” on acute inflammation induced in mice and analyze the underlying mechanisms. The study aimed to utilize the anti-inflammatory effects of CBD on acute inflammation in mice as foundational research data for potential applications in human acute inflammation. As a result, the study intended to prove the necessity of the cultivation of “pink pepper” with a high CBD content and establish the value of the material. We investigated the anti-inflammatory effect and determined the industrial value of CBD isolated from “Pink Pepper”.

## 2. Results

### 2.1. Cell Viability of CBD in RAW 264.7 Cells

We examined the effect of CBD on RAW 264.7 cells using a CCK-8 kit to confirm cell viability of CBD. Cells were treated with CBD at concentrations of 1.25, 2.5, and 5 μM. Consequently, compared to the control group, the CBD-treated cells did not show changes in the concentrations of 1.25 μM, 2.5 μM, and 5 μM. No toxicity was observed at the maximum concentration of 5 μM (Figure 1B).

### 2.2. CBD Inhibited the Overproduction of Nitric Oxide in LPS-Induced RAW 264.7 Cells

**Figure 2:**
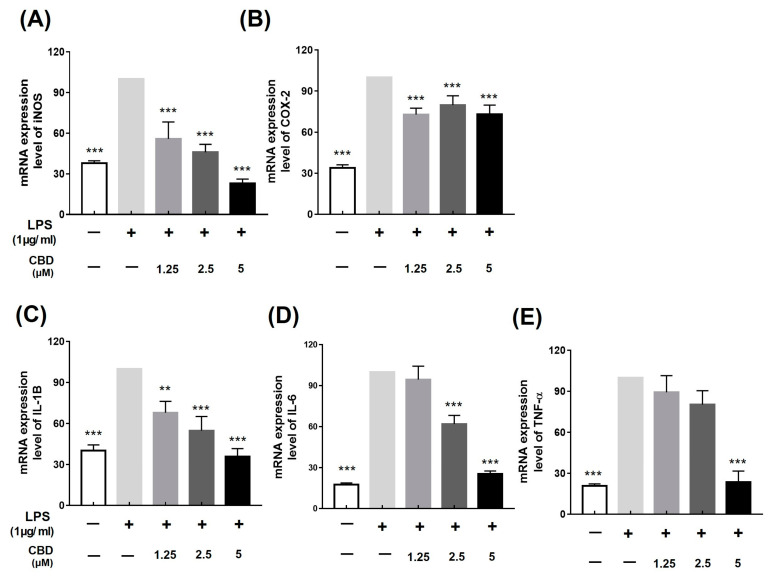
Inhibitory effect of CBD on mRNA expression of inflammatory factors. All samples except control were treated with 1 μg/mL of LPS. (**A**) iNOS; (**B**) COX-2; (**C**) IL-1β; (**D**) IL-6; and (**E**) TNF-α mRNA expression levels in groups treated with various concentrations of CBD compared with those in the LPS-only treatment group. Data represent the means ± SD in triplicate. *p*-value was calculated based on LPS-only data using ANOVA and Tukey’s post hoc test (** *p* < 0.01, *** *p* < 0.001).

LPS-treated macrophage RAW 264.7 cells were treated with CBD at concentrations of 1.25, 2.5, and 5 μM, and NO overproduction was measured. The NO production rate was higher in the LPS-treated group than in the control group (13.8 ± 0.66%). In addition, when CBD was administered at concentrations of 1.25, 2.5, and 5 μM, the NO production rate was 93.7 ± 2.56%, 78.5 ± 1.58%, and 72.52 ± 1.84%, respectively (Figure 2B). Therefore, we confirmed that CBD reduced LPS-induced NO overproduction in a concentration-dependent manner (Figure 1C).

### 2.3. The Effect of CBD on the Transcription Inactivation of Inflammatory Genes

We examined the effect of CBD on the production of inflammatory cytokines and the transcription of genes related to inflammation. The inflammatory genes’ mRNA was not expressed in the LPS-untreated control. mRNA expression was inhibited by 55.80 ± 10.18%, 45.97 ± 4.76%, and 22.96 ± 2.64 in the case of iNOS according to concentrations of 1.25, 2.5, and 5 μM of CBD, respectively (Figure 2A). In the case of COX-2, mRNA expression was suppressed by 73.02 ± 5.51% at the highest concentration of 5 uM CBD, however, not in a concentration-dependent manner (Figure 2B). IL-1β, TNF-α, and IL-6, which are important cytokines for inflammatory regulation, decreased in expression by 35.86 ± 4.73% (1.25 μM), 25.38 ± 1.80% (2.5 μM), and 23.69 ± 6.50% (5 μM) (Figure 2C–E). CBD was shown to inhibit the mRNA expression of iNOS, IL-1β, TNF-α, and IL-6 in a concentration-dependent manner (Figure 2).

### 2.4. CBD Inhibited the Increase in iNOS and Inflammatory Cytokines Induced by LPS Treatment

**Figure 3:**
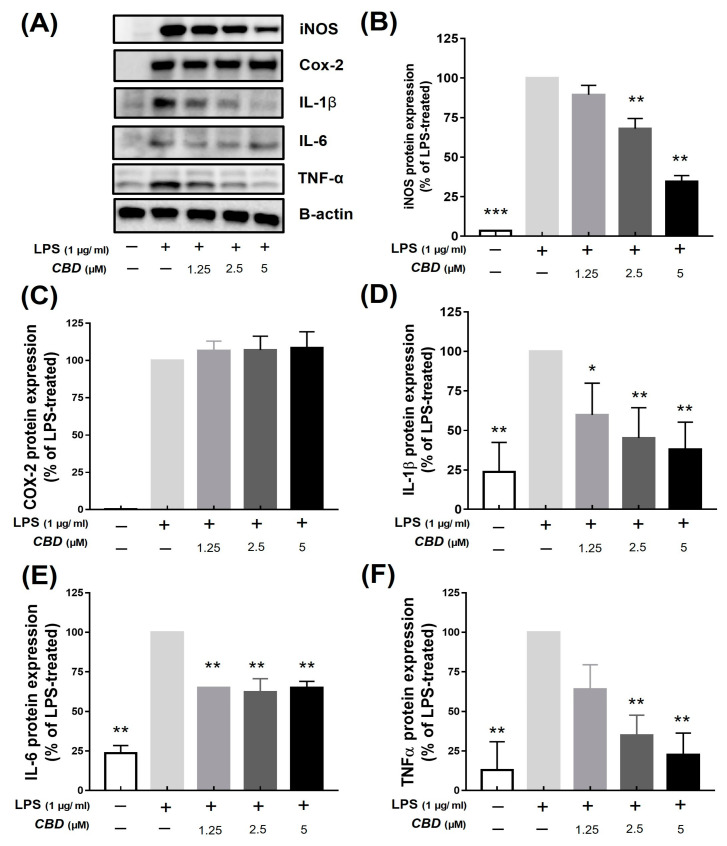
Inhibitory effect of CBD on protein expression of inflammatory factors. All samples except control were treated with 1 μg/mL of LPS. (**A**) Western blot detection bands of each inflammatory factor protein in groups treated with various concentrations of CBD compared with that of (**B**) iNOS; (**C**) COX-2; (**D**) IL-1β; (**E**) IL-6; and (**F**) TNF-α protein expression levels in LPS-only treatment group. *p*-value was calculated based on LPS-only data using ANOVA and Tukey’s post hoc test (* *p* < 0.05, ** *p* < 0.01, *** *p* < 0.001).

The protein expression of iNOS was inhibited by 89.45 ± 4.17%, 68.06 ± 4.49%, and 34.47 ± 2.76% in cells treated with 1.25, 2.5, and 5 μM of CBD, respectively (Figure 3B). The protein expression of COX-2 did not change in cells treated with 5 μM CBD compared to the LPS-only treatment group (Figure 3C). The protein expression of IL-1β decreased by 59.80 ± 17.38%, 45.18 ± 16.61%, and 37.88 ± 15.02% in cells treated with 1.25, 2.5, and 5 μM of CBD, respectively (Figure 3D). In contrast, the protein expression of IL-6 decreased in cells treated with 1.25 μM CBD (64.93 ± 0.19), and no concentration-dependent changes were observed (Figure 3E). The protein expression of TNF-α decreased in a concentration-dependent manner by 63.92 ± 13.46% (1.25 μM), 34.91 ± 10.97% (2.5 μM), and 22.66 ± 11.09% (5 μM) in cells treated with CBD (Figure 3F). The results for iNOS and cytokines, but not those for COX-2, were consistent with the mRNA results, suggesting that CBD reduced the expression of LPS-induced inflammatory proteins. 

### 2.5. CBD Regulates Inflammation by Inhibiting MAPK Pathway

**Figure 4:**
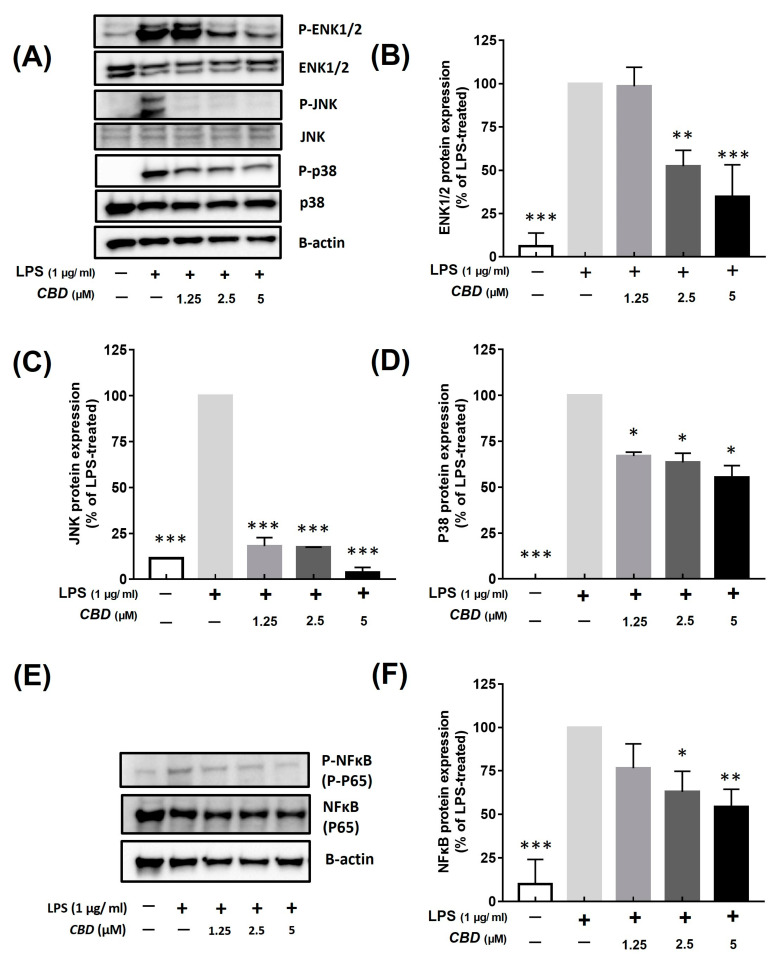
Inhibitory effect of CBD on phosphorylation of MAPK and NF-κB signaling pathways. All samples except control were treated with 1 μg/mL of LPS. (**A**) Western blot detection bands of each MAPK protein in groups treated with various concentrations of CBD compared with that of (**B**) p-ERK1/2; (**C**) p-JNK; and (**D**) p-P38 in LPS-only treatment group. (**E**) Western blot detection bands of NF-κB protein in groups treated with various concentrations of CBD compared with that of (**F**) p-NF-κB protein in LPS-only treatment group. *p*-value was calculated based on LPS-only data using ANOVA and Tukey’s post hoc test (* *p* < 0.05, ** *p* < 0.01, *** *p* < 0.001).

We confirmed the effects of CBD on the JNK, ERK, and P38 proteins, which are important factors in the MAPK pathway. MAPK was not phosphorylated in the LPS-untreated control group. ERK1/2 was inhibited by 98.48 ± 9.90%, 52.42 ± 7.90%, and 34.76 ± 15.96% at concentrations of 1.25, 2.5, and 5 μM of CBD, respectively (Figure 4B). JNK was inhibited in the LPS-untreated control group by 18.06 ± 3.33%, 17.47 ± 0.05%, and 3.63 ± 1.99% at CBD concentrations of 1.25, 2.5, and 5 μM, respectively (Figure 4C). Moreover, the phosphorylation of P38 was inhibited by 66.95 ± 1.50%, 63.52 ± 3.56%, and 55.27 ± 4.60% depending on each concentration of CBD (Figure 4D). We found that CBD suppressed physiological responses and blocked inflammatory factors by inhibiting MAPK phosphorylation (Figure 4A).

### 2.6. CBD Inhibits the Phosphorylation of NF-κB Stimulated by LPS

We confirmed the effect of CBD on B phosphorylation, which plays an important role in cytokine and inducible enzyme expression. NF-κB was not phosphorylated in the LPS-untreated control group. The phosphorylation of NF-κB was inhibited in the LPS-untreated group by 76.50 ± 12.16%, 62.79 ± 10.33%, and 54.19 ± 8.80% at CBD concentrations of 1.25, 2.5, and 5 μM, respectively (Figure 4E,F).

### 2.7. CBD Inhibits the Acute Inflammatory Response Induced by λ-Carrageenan in Mouse

**Figure 5:**
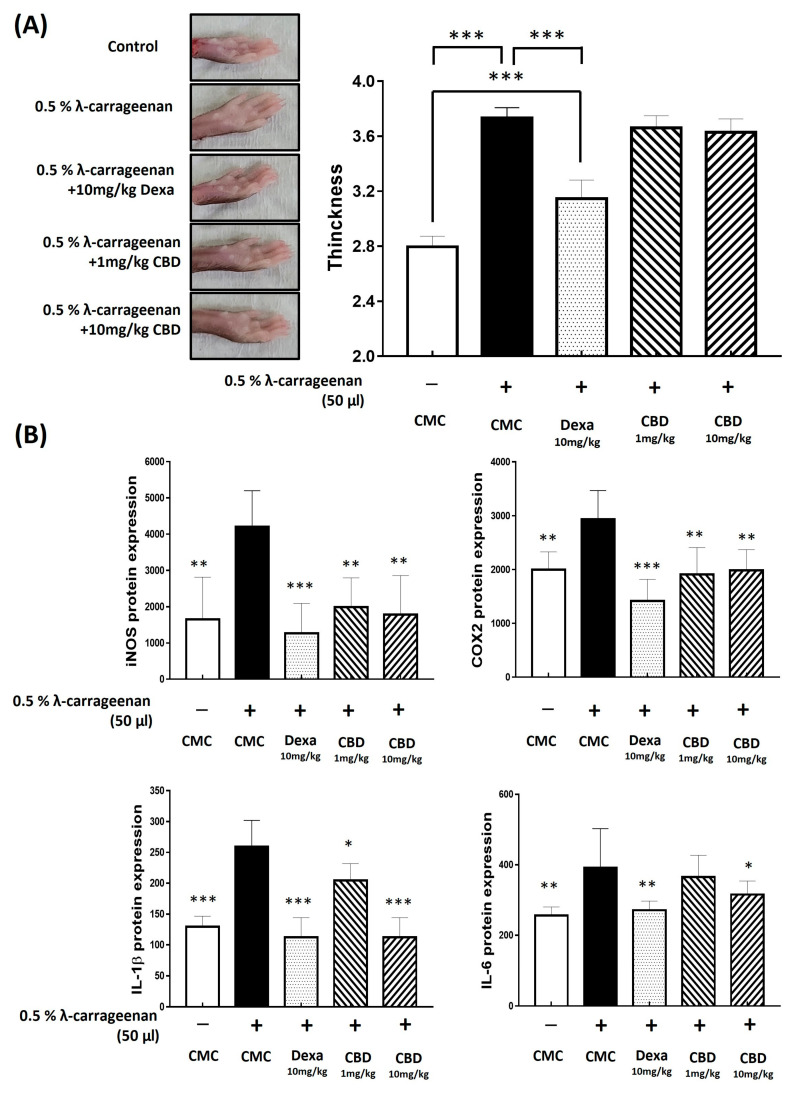
Effects of CBD on λ-carrageenan-induced edema in mice. Paw edema and inflammatory factor production in mice induced by λ-carrageenan. (**A**) Paw edema was measured using calipers 4 h after λ-carrageenan administration and compared with that in the normal group. (**B**) Inflammatory factor production was assessed using enzyme-linked immunosorbent assay (ELISA). *p*-value was calculated based on LPS-only data using ANOVA and Tukey’s post hoc test (* *p* < 0.05, ** *p* < 0.01, *** *p* < 0.001).

The in vivo anti-inflammatory effect of CBD was confirmed using a model in which acute inflammation was induced in mice with 0.5% λ-carrageenan. Edema tissue continued to develop after treatment with 0.5% λ-carrageenan and orally administered CBD in mice. Both high (10 mg/kg) and low concentrations (1 mg/kg) showed no change compared to the 0.5% λ-carrageenan alone treatment group. In contrast, the group treated with a positive control dexamethasone 10 mg/kg showed a significant difference compared to the group that received a 0.5% λ-carrageenan-only injection 4 h after the injection of 0.5% carrageenan (Figure 5). Proteins were extracted from the paws of mice treated with 0.5% λ-carrageenan, and inflammatory factors were analyzed using ELISA. iNOS was inhibited by 4204.57 ± 921.55 pg, 1991.50 ± 750.82 pg, and 1778.00 ± 1025.75 pg according to the concentrations of 0, 1, and 10 mg/kg of CBD, respectively (Figure 5B). In the group injected with 0.5% λ-carrageenan only, COX-2 was increased to 2934.31 ± 498.91 pg, and oral administration of 1 mg/kg and 10 mg/kg of CBD inhibited COX-2 to 1908.61 ± 466.27 pg and 1985.87 ± 353.93 pg (Figure 5B). Through oral administration of CBD at doses of 1 mg/kg and 10 mg/kg, IL-1β overexpression induced by a 0.5% λ-carrageenan injection was suppressed from 259.38 ± 39.65 pg to 204.46 ± 25.56 pg and 112.50 ± 29.53 pg, respectively (Figure 5B). No significant decrease in IL-6 was observed at low CBD concentrations. However, at a high concentration of CBD 10 mg/kg, the IL-6 that increased to 391.61 ± 104.57 pg through the 0.5% λ-carrageenan injection was reduced to 315.24 ± 36.34 pg (Figure 5B). 

These results indicated that CBD suppressed the inflammatory response in mice with acute inflammation. CBD administration did not significantly reduce the size of the edematous tissue; however, it resulted in a significant decrease in the production of inflammatory factors in the mouse paw edema tissue (Figure 5).

## 3. Discussion

Inflammation is one of the defense mechanisms of the body and is usually initiated by cellular damage from internal or external tissue sources [32]. Chronic and excessive inflammatory reactions lead to the development of acute and chronic inflammation in various organs, including the liver, kidney, lung, brain, cardiovascular, and reproductive organs. These reactions result in impaired functioning of cells or tissues and genetic mutations and contribute to the onset of numerous diseases [32]. As inflammation involves many complexes signaling pathways that are tightly regulated, uncontrolled, and unresolved, chronic inflammation can be detrimental to the host [32].

To evaluate the anti-inflammatory efficacy of CBD, we confirmed whether it inhibits the expression of inflammatory factors using LPS-induced RAW 264.7 cells. When CBD was administered at a concentration of 5 μM, a cell viability of at least 80% was maintained (Figure 1B). Therefore, we used concentrations of up to 5 μM in subsequent experiments to confirm nitric oxide (NO) production. We found that CBD inhibited NO production, and a treatment of 5 μM CBD decreased the LPS-induced increased protein expression of iNOS, IL-1β, IL-6, and TNF-α (Figure 1, Figure 2 and Figure 3). However, CBD treatment did not affect the protein expression of COX-2 (Figure 3C). Unlike iNOS, which mediates nitric production, COX-2 follows a pathway that increases prostaglandin E2 (PGE2) levels, thereby inducing inflammatory responses [33,34]. The inhibition of inflammation using selective inhibitors of iNOS and COX-2 has been reported to be specific and pharmacologically significant [35,36]. We confirmed that COX-2 shows a limited response to CBD, and CBD effectively reduces inflammatory mediators, such as iNOS, and cytokines, such as IL-1β, IL-6, and TNF-α. These results suggest the potential utility of CBD in treating various conditions related to both chronic and acute inflammatory responses.

Nitric oxide (NO) is an effector of the innate immune system and is produced by iNOS. Increased NO levels due to iNOS overexpression promote atherosclerosis, tumor growth, and apoptosis [37,38]. The overexpression of cytokines IL-1β, IL-6, and TNF-α is known to affect systemic acute inflammatory syndrome, chronic immune disease, pain, cardiovascular disease, and diabetes [39,40,41]. In addition, Schultheiß et al. reported that the overexpression of IL-1β, IL-6, and TNF-α may be a crucial factor in the postacute sequelae of SARS-CoV-2. Our results suggest that CBD can act as an anti-inflammatory agent and could potentially be used against various diseases [42].

Cannabidiol (CBD) is a small-molecule phytocannabinoid comprising a pentyl-substituted bisphenol aromatic group (pentylresorcinol) linked to an alkyl-substituted cyclohexene terpene ring system (Figure 1A) [28]. CBD has multiple pharmacological effects and has been extensively tested against various diseases [28]. Moreover, CBD acts as an agonist and antagonist of many receptors. The receptors to which cannabinoids typically bind include CB_1_ and CB_2_ receptors [43]. The CB_1_ receptor is also expressed in the heart, liver, pancreas, muscle, adipose tissue, and reproductive system [43]. CB_2_ receptors are primarily expressed in cells associated with the immune system, such as white blood cells; however, they are also found in the spleen, thymus, bone marrow, and other tissues involved in immune functions [43]. CBD does not directly bind to CB_1_ and CB_2_ receptors but acts indirectly through allosteric binding activity [43,44]. In addition, TRPV receptors and PPARγ receptors are strong molecular targets of CBD. Possibly, CBD could also bind to other receptors [29,43]. The affinity for various receptors of CBD explains its multifaceted pharmacological characteristics [29,44], suggesting the potential of CBD as an economical material that can target not only anti-inflammatory effects but also multiple effects simultaneously. 

The CB_1_ receptor and CB_2_ receptor are known to regulate the MAPK pathway [45,46,47] and participate in apoptosis through NF-κB [48]. In addition, among the TRPV receptors, which are strong target receptors of CBD, TRPV1 and TRPV4 are also known to regulate the MAPK and NF-κB pathways [49,50]. Herein, treatment with 5 μM CBD decreased the LPS-induced increased protein expression of p44/42 MAPK (ERK1/2), JNK, and P38 and suppressed NF-κB phosphorylation, confirming that CBD inhibited the MAPK and NF-κB pathways (Figure 4). The reduction in MAPK and NF-κB in macrophages is thought to be the most important target for their anti-inflammatory effects [51,52]. Therefore, we conjectured that CBD produces anti-inflammatory effects by regulating the MAPK and NF-κB pathways by acting on their receptors. However, to decide on this mechanism, further investigations are required to ascertain whether CBD is applicable at concentrations relevant to receptors and whether it permeates the cell membrane within an applicable timeframe. In addition, as the MAPK and NF-κB pathways are involved not only in inflammatory responses but also in various physiological reactions, further research is needed to explore the potential positive and negative impacts on different biological responses related to these pathways.

λ-carrageenan, a mucopolysaccharide extract, has long been used as a stimulant in local inflammation models to induce local edema, leukocyte infiltration, and local PGE2 production [53,54]. We induced an acute inflammatory response by injecting λ-carrageenan into the right hind paw of rats and confirmed the anti-inflammatory effect of oral CBD administration (Figure 5). Although CBD treatment did not affect the thickness of the acutely inflamed paw tissue, it resulted in changes in the protein expression of inflammatory factors in the inflamed paw tissue. A concentration-dependent decrease in the levels of iNOS, COX-2, and cytokines (IL-1β and IL-6) was observed in λ-carrageenan-treated groups. A concentration-dependent decrease in the levels of iNOS, COX-2, and cytokines (IL-1β and IL-6) was observed in the λ-carrageenan-treated groups. However, CBD treatment did not affect the thickness of the acutely inflamed paw tissue, suggesting that the changes we observed at the protein level were not expressed at the tissue level. Furthermore, CBD elicited a weak response in COX-2 inhibition, suggesting that the local anti-inflammatory effects of CBD may not be sufficient to respond to the λ-carrageenan-induced acute inflammatory response. Another possibility is that CBD has considerable drawbacks owing to its slow absorption and extensive inactivation through first-pass metabolism when administered orally [44]. A report indicated that when CBD was administered locally rather than orally, it accumulated more in the skin and muscles, confirming safe treatment results [44,55]. In addition, oral administration or when administered in combination with THC or other substances of CBDA, a strongly acidic precursor of CBD, has a stronger efficacy compared with that of CBD administered alone [56]. Our results suggest that CBD showed anti-inflammatory efficacy in the acute inflammatory mouse model induced by λ-carrageenan. However, no tissue effect was observed, suggesting that its efficacy may be weakened when administered orally. Therefore, care should be taken when using CBD.

In addition to considerations regarding these administration routes, there are limitations to research involving CBD. The most prominent among these is psychological resistance and concerns about misuse. Cannabis remains associated with powerful addiction and misuse globally, having been controlled due to its psychoactive nature [57]. According to the United Nations Office on Drugs and Crime, it is the most widely used illicit drug in the 21st century [57]. Even though CBD doe not have a psychoactive nature, there is psychological resistance due to its origin from cannabis. Also, there are concerns internationally that CBD usage might lead to a positive portrayal of the use of cannabis, and illegal consumption could increase [57]. And, the safety of CBD has not been fully evaluated, and its effects have not been accurately delineated [58]. Research on the side effects of CBD is limited and tends to focus on positive outcomes only. However, the multifaceted medical effects of CBD cannot be disregarded, and regulations are rapidly evolving in many countries to embrace the potential benefits of medical hemp and CBD. With this interest, the potential of hemp and CBD for medical use should be appropriately studied in various aspects, especially the need to study the biggest issue, the route of administration and carefully controlled dose effects.

## 4. Materials and Methods

### 4.1. Reagents

RAW 264.7 cells were purchased from the American Type Culture Collection (ATCC, Rockville, MD, USA). Pentane, ipopolysaccharide, and λ-carrageenan were purchased from Sigma-Aldrich (St Louis, MO, USA), and DMEM cell culture media, fetal bovine serum (FBS), penicillin, and streptomycin were purchased from Gibco BRL (Invitrogen Co., Carlsbad, CA, USA). Cell Counting Kit-8 (CCK-8) was purchased from Dojindo Molecular Technologies (Rockville, MD, USA). The NO detection kit and PRO-PREP™ were purchased from iNtRON (Seongnam, Republic of Korea). Absorbance was measured using a device purchased from Molecular Devices (Sunnyvale, CA, USA). The RNA isolation kit was purchased from Qiagen (Santa Clarita, CA, USA). The reverse transcription master mix was purchased from Elpis Biotech (Daejeon, Republic of Korea). All primary antibodies, iNOS, COX2, TNF-α, IL-6, IL-1β, Erk1/2, p-Erk1/2, JNK, P-JNK, P38, p-P38, NFκB, P-NFκB, and β-actin, were purchased from Cell Signaling (Denver, MA, USA). iNOS and COX-2 enzyme-linked immunosorbent assay (ELISA) kits were purchased from MyBioSource (San Diego, CA, USA), and the IL-6 and IL-1β ELISA kits were purchased from R&D Systems (Minneapolis, MN, USA).

### 4.2. Sample Preparation

The hemp (*Cannabis sativa* ssp. *sativa*) used in this experiment was the inflorescence of pink pepper (GenBank accession number: GCA_029168945.11). The pink pepper was cultivated by the Chuncheon Bioindustry Foundation (CBF, Chuncheon, Republic of Korea: 37°53′33″ N, 127°44′38″ E) and was newly developed by Lim (2022) as a new variety [14,15]. We dried the pink pepper using a hot air dryer (Daedong, Daegu, Republic of Korea) for 48 h at 40 °C. After drying, it was pulverized to a size of 80 mesh using a grinder (DA280-S, Daesung, Paju, Republic of Korea) and stored at a temperature of 23 °C and humidity of 14% in a thermo-hygrostat (DH.DeADDBG1K, Daihan, Wonju, Republic of Korea).

### 4.3. Supercritical Fluid Extraction (SFE) Procedure

A total of 1000 g of dry hemp sample was placed in a 10 L supercritical extractor container. The container was sealed to avoid gas leakage, and the supercritical extractor was utilized for 110 min with a CO_2_ flow of 500 g/min, sampler temperature of 54 °C, separator temperature of 30 °C, and pressure of 472.7 bar (SFE-CO2-10L-40M-60C-R, Phos-enthech, Daejeon, Republic of Korea). The collected extract was left at room temperature for approximately 2 h until the carbon dioxide evaporated, and the weight of the extract was measured.

### 4.4. CBD Purification

To produce high-purity CBD from the crude oil extracted from the supercritical extractor, 99% ethanol was added to 10 times the amount of the extract, and the sample was stored in a deep freezer (Thermo Fisher Scientific, Waltham, MA, USA) at −70 °C for 24 h. To separate the solidified wax, the solution was filtered through a vacuum filter and filter paper (WF2-0900, Whatman, Maidstone, UK). Ethanol of the filtered extract was evaporated in a vacuum concentrator (Rotavapor, R-220SE, Büchi Labortechnik AG, Flawil, Switzerland) at 70 bar and 45 °C for 60 min, and CBD and CBD-A were isolated through medium pressure liquid chromatography (SIO-1EV, Biotage, Uppsala, Sweden). After removing the solvent from the separated CBD and CBD-A using a vacuum concentrator, they were reduced for 50 min at 125 °C in an oven (S-30; Saehan, Seoul, Republic of Korea) to convert CBD-A to CBD.

### 4.5. CBD Crystallization

To convert purified high-purity CBD from a liquid to solid state, 0.5 mL of pentane was added per 1 g of sample, melted at 30 °C, placed in a beaker, and stored in a freezer at −4 °C for 10 min. The solution was stirred at 120 RPM using a magnetic stirrer, washed with pentane cooled to −5 °C, and dried at room temperature for 30 min using a vacuum dryer (Ilshinbiobase Co., Ltd., Yangju, Republic of Korea) to remove the solvent from the crystallized CBD.

### 4.6. Cell Culture and Cell Viability Assay

RAW 264.7 cells were maintained in Dulbecco’s Modified Eagle medium (DMEM) supplemented with 10% fetal bovine serum (FBS), 1% penicillin, and streptomycin. Cells were grown at 37 °C in 5% CO_2_. The proliferation and viability of RAW 264.7 cells after sample treatment were confirmed through the Cell Counting Kit-8 (CCK-8) assay. The cells were seeded at 1 × 10^4^ cells/well in 96-well cell culture plates and cultured for 18 h. Then, CBD sample solutions at various concentrations (1.25, 2.5, and 5 μM) were, respectively, added for 24 h. The CCK-8 solution (10 μL/well) was added to the cells in 96-well plates and measured according to the manufacturer’s protocol.

### 4.7. Nitric Oxide (NO) Assay

To measure anti-inflammatory activity, RAW 264.7 cells were seeded at 2 × 10^5^ cells/well in 24-well plates and incubated for 24 h. Subsequently, the cells were treated with LPS at a concentration of 1 μg/mL. After 2 h of LPS treatment, samples were treated with each respective concentration of CBD (1.25, 2.5, and 5 μM) for 18 h. Thereafter, nitrite oxide content in the supernatant was evaluated using a NO detection kit, and absorbance was measured at 560 nm using a plate reader (Molecular Devices, Sunnyvale, CA, USA).

### 4.8. Reverse Transcription-Quantitative PCR (RT-qPCR)

For the evaluation of inflammation-related factors through mRNA expression levels, RNA was extracted from the RAW264.7 cells (2.5 × 10^5^ cells/mL) using an RNA isolation kit. RNA (1 μg), the purity of which was measured using the NanoDrop (Thermo Fisher Scientific, Madison, WI, USA), was synthesized into cDNA using the reverse transcription master mix. Relative expression levels were analyzed using qPCR using oligonucleotide primers (Table 1) and measured using a LightCycler 480 (Roche, Mannheim, Germany). The qPCR conditions were as follows: 95 °C for 5 min, followed by 40 cycles of 95 °C for 15 s, 58 °C for 15 s, and 72 °C for 30 s, normalized to GAPDH for relative quantitative analysis.

### 4.9. Preparation of Cell Lysates and Nuclear Fractions and Western Blot Analysis

RAW 264.7 cells were seeded in a 6-well plate at 2.5 × 10^5^ cells/well and cultured for 18 h, after which they were treated with 1 μg/mL LPS and further cultured for 2 h. Cells were treated with each sample concentration of CBD (1.25, 5, and 2.5 μM) for 24 h. Total protein was extracted using PRO-PREP™, and protein concentration was measured using the Pierce™ BCA Protein Assay (Thermo Fisher Scientific, Rockford, IL, USA). 

The extracted proteins were separated by size using SDS-PAGE, transferred to a membrane, and blocked with 5% BSA. Protein concentration-dependent luminescence using SuperSignal Western Blot Enhancer (Pierce, Rockford, IL, USA) was also confirmed using LAS-4000 (Fujifilm, Tokyo, Japan).

### 4.10. λ-Carrageenan-Induced Paw Edema Mouse Model

In this study, 6–7-week-old C57BL/6 male mice (Central Lab. Animal Inc., Seoul, Republic of Korea) were used after stabilization for 7 d. We raised 3 to 4 experimental animals in one polycarbonate cage and provided spruce bedding (Bedding FS14, safe Lab, Rosenberg, Germany) of 3.5 mm to 4.5 mm in size. The maintained conditions were as follows: free access to food and water, temperature of 23 ± 2 °C, humidity of 55% ± 5%, and a 12 h light/dark cycle. CBD was administered orally at doses of 1 or 10 mg/kg for a total of four days (three days before and on the day of carrageenan injection). To induce acute paw inflammation, 0.5% λ-carrageenan (50 μL) was subcutaneously injected into the right hind paw; sterile saline (50 μL) was injected into the control group. 

### 4.11. Measurement of Production of Inflammatory Factors in Mouse Paw Tissue

To extract proteins from tissues of 0.5% λ-carrageenan-induced paw edema models, the tissues were frozen in liquid nitrogen, pulverized using a mortar, and lysed in PRO-PREP™. The lysed tissue was centrifuged at 13,000 rpm for 15 min at 4 °C to separate the supernatant. The separated supernatant was subjected to ELISA in accordance with the manufacturer’s protocol to confirm and measure iNOS, COX-2, IL-1β, and IL-6 levels using the following kits: iNOS ELISA kit (MBS261100, mybiosource, BC, CA), COX-2 ELISA kit (MBS269104, mybiosource, BC, CA), IL-1β ELISA kit (MLB00C, R&D Systems, USA), IL-6 ELISA kit (M6000B, R&D Systems, USA).

### 4.12. Data Analysis

All experiments were conducted as independent experiments and were repeated at least thrice. All results were expressed as mean ± standard deviation (mean ± SD). The experimental results were graphed using GraphPad Prism (GraphPad Software 8.0.1), and statistical analysis of each experimental result was performed using one-way analysis of variance (ANOVA), validated at the 5%, 1%, and 0.1% levels of significance (* *p* < 0.05, ** *p* < 0.01, *** *p* < 0.001). 

## 5. Conclusions

**Figure 6:**
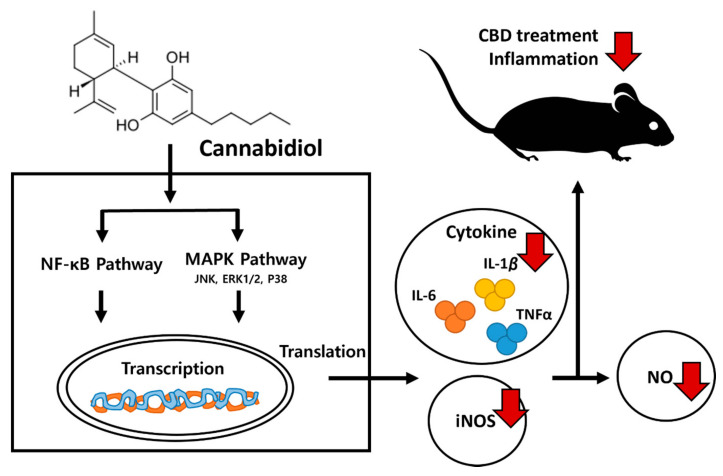
Proposed mechanism of inhibition of inflammatory signaling by CBD.

CBD has several physiological implications, and multiple medical effects are attracting attention amid social and research concerns. This study showed CBD has strong anti-inflammatory effects (Figure 6). The inhibitory effect of CBD on anti-inflammatory markers in vitro and in vivo highlighted in this study can be used as a baseline for the use of CBD as an anti-inflammatory agent. Our findings suggest that CBD can be used to treat various diseases associated with chronic and acute inflammatory responses. Therefore, the newly developed “Pink pepper” cultivar with a high CBD content has sufficient industrial value and excellent pharmacological and physiological value.

## Figures and Tables

**Table 1:** Primer of inflammatory concern gene used in RT-PCR analysis in this study.

Genes	Forward Primer (5′ → 3′)	Reverse Primer (5′ → 3′)
iNOS	AATGGCAACATCAGGTCGGCCATCACT	GCTGTGTGTCACAGAAGTCTCGAACTC
COX2	GGAGAGACTATCAAGATAGT	ATGGTCAGTAGACTTTTACA
IL-1β	TGCAGAGTTCCCCAACTGGTACATC	GTGCTGCCTAATGTCCCCTTGAATC
IL-6	GAGGATACCACTCCCAACAGACC	AAGTGCATCATCGTTGTTCATACA
TNF-α	ATGAGCACAGAAAGCATGATC	TACAGGCTTGTCACTCGAATT
GAPDH	GTATGACTCCACTCACGGCAAA	GGTCTCGCTCCTGGAAGATG

## Data Availability

All data are contained within the article.

## Sample Availability

Samples of the CBD isolated from “Pink Pepper” are available from the corresponding authors.
